# ACPA-negative RA consists of subgroups: patients with high likelihood of achieving sustained DMARD-free remission can be identified by serological markers at disease presentation

**DOI:** 10.1186/s13075-019-1902-2

**Published:** 2019-05-14

**Authors:** Debbie M. Boeters, Leonie E. Burgers, Eric H. Sasso, Tom W. J. Huizinga, Annette H. M. van der Helm – van Mil

**Affiliations:** 1 Department of Rheumatology, Leiden University Medical Center, PO Box 9600, 2300 RC Leiden, the Netherlands; 2 Crescendo Bioscience, South San Francisco, CA USA; 3 Department of Rheumatology, Erasmus University Medical Center, Rotterdam, the Netherlands

**Keywords:** Rheumatoid arthritis, ACPA, Sustained DMARD-free remission, Biomarker

## Abstract

**Background:**

Disease-modifying antirheumatic drug (DMARD)-free remission, the sustained absence of synovitis after DMARD cessation, is increasingly achievable, especially in autoantibody-negative rheumatoid arthritis (RA). However, underlying mechanisms are unknown and patient subgroups that achieve this outcome are insufficiently characterized. We evaluated whether serological biomarkers at disease onset, as measured within the multi-biomarker disease activity (MBDA) score, are differently expressed in RA patients who achieve sustained DMARD-free remission.

**Methods:**

Two hundred ninety-nine RA patients were evaluated for achievement of sustained DMARD-free remission during a median follow-up of 4.3 years. Twelve biomarkers, as included in the MBDA score, were determined from the serum obtained at disease onset. Patients were categorized as having a low (< 30), moderate (30–44) or high (> 44) score. Analyses were stratified for anti-citrullinated protein antibodies (ACPA) based under the assumption that ACPA-positive and ACPA-negative RA are different disease entities.

**Results:**

Twenty percent achieved sustained DMARD-free remission. Overall, high MBDA scores were associated with achieving DMARD-free remission (high vs. low HR 3.8, 95% CI 1.2–12.2). Among ACPA-negative RA patients, moderate or high scores associated strongly with DMARD-free remission (moderate vs. low HR 9.4, 95% CI 1.2–72.9; high vs. low HR 9.7, 95% CI 1.3–71.1). This association was independent of age and other clinical factors (high vs. low HR 8.2, 95% CI 1.1–61.8). For ACPA-negative RA patients, the biomarkers C-reactive protein, serum amyloid A and matrix metalloproteinase-3 were individually associated with sustained DMARD-free remission. Among ACPA-positive RA patients, scores were not associated with DMARD-free remission.

**Conclusions:**

ACPA-negative RA patients who achieved sustained DMARD-free remission after treatment withdrawal were characterized by moderate to high MBDA scores at diagnosis. This is the first evidence that ACPA-negative RA can be subdivided in clinically relevant subsets at disease onset using a protein profile.

**Electronic supplementary material:**

The online version of this article (10.1186/s13075-019-1902-2) contains supplementary material, which is available to authorized users.

## Background

Rheumatoid arthritis (RA) is a syndrome which presumably consists of several disease entities. Most data have focused on differences in RA characterized by the presence and absence of autoantibodies, in particular anti-citrullinated protein antibodies (ACPA). ACPA-positive patients have in general a more persistent and destructive disease course than ACPA-negative patients. The generation of different disease subsets in seronegative patients that have a clinical diagnosis of RA and fulfil respective classification criteria is unsuccessful thus far [1]. Therefore, we investigated if we could identify patients in the ACPA-negative subgroup that have the best clinical outcome, which currently is the achievement of sustained disease-modifying antirheumatic drug (DMARD)-free remission.

The biological mechanisms underlying the achievement of sustained DMARD-free remission are unknown. Additionally, it is undefined whether this outcome is potentially achievable by all RA patients or whether the ability to permanently stop DMARDs is restricted to a set of RA patients with certain biological characteristics. Several studies have shown that a shorter symptom duration, which is a disease phase characteristic rather than a ‘patient characteristic’, is associated with a greater probability of achieving sustained DMARD-free remission [2–6]. The second important factor is the absence of ACPA [1, 2, 6]. This suggests that patients who can achieve remission are inherently different. However, the absence of autoantibodies only explains part of the variability in outcome, since a proportion of ACPA-positive patients can achieve sustained DMARD-free remission and the majority of ACPA-negative patients do not achieve it [7]. We assumed that patients who are able to achieve sustained DMARD-free remission are intrinsically different from patients who are unable to do so. If this hypothesis is true, these patients might be identifiable by biomarkers present at disease presentation. With respect to systemically measurable markers, C-reactive protein (CRP) has been studied and increased levels were associated with sustained DMARD-free remission in one study [2], while in another study no association was observed [8]. Other inflammatory proteins have not been studied in relation to sustained DMARD-free remission.

Several serological biomarkers are combined in the multi-biomarker disease activity (MBDA) score, which is developed to measure RA disease activity [9, 10]. The level of the 12 biomarkers which are combined in the MBDA score might indicate relevant pathways involved in RA disease activity, and the combination of markers may provide more information than markers such as the erythrocyte sedimentation rate (ESR) or CRP alone. Several studies have shown that higher MBDA scores measured during the disease course are predictive of radiographic progression in the next years [11–13], although there are also studies showing no association [14–16]. It is unexplored if the serological biomarkers included in the score are associated with an opposite, favourable outcome, i.e. achieving sustained DMARD-free remission.

Our ultimate aim was to identify subgroups of RA patients that are identifiable at disease presentation, for which sustained DMARD-free remission is an achievable outcome. We hypothesized that individual serological markers or a combination of these is helpful to characterize these subgroups. Therefore, we investigated the association between the MBDA score and its component serological markers at first presentation with RA and the achievement of sustained DMARD-free remission. We observed that the subgroup of ACPA-negative RA patients with a high chance of achieving sustained DMARD-free remission can already be identified at the time of diagnosis by the presence of a combination of proteins.

## Methods

### Patients

The Leiden Early Arthritis Clinic cohort is an inception cohort that enrolls patients with clinically confirmed arthritis of recent onset and symptom duration < 2 years. At baseline, questionnaires were administered, joint counts and blood samples were collected and patients were evaluated annually thereafter [1]. Baseline serum samples were tested for CRP level, ESR, IgG ACPA (EliA CCP (anti-CCP2), Phadia, Nieuwegein, the Netherlands) and IgM rheumatoid factor (RF; in-house ELISA, as described previously [17]). Patients did not use DMARDs or glucocorticoids before inclusion.

For this study, RA patients included between 2010 and 2015 were evaluated, since this is the most recent inclusion period and since we have shown that sustained DMARD-free remission is increasingly achievable with current treatment strategies [8]. RA was stringently defined by a clinical diagnosis of RA by an experienced rheumatologist. Besides a clinical diagnosis, patients needed to fulfil the 1987 or 2010 classification criteria during the first year [18, 19]. Both classification criteria were considered since ACPA-negative patients can be misclassified by the 2010 criteria because they need > 10 involved joints to achieve 6 points. Thus, all included RA patients had a clinical diagnosis of RA and in addition fulfilled RA classification criteria. Patients diagnosed with conditions other than RA during the follow-up were not included in this study. In the period mentioned, 321 patients were eligible. Thirteen patients were excluded because they did not use DMARDs during the follow-up and 9 because measurement of an MBDA biomarker had failed. Thus, in total, 299 patients were studied.

The initial treatment of RA consisted of methotrexate, which could be combined with low-dose prednisone bridging therapy at DMARD start. Typically, when the first treatment failed, another conventional DMARD was initiated or added. A biological DMARD was allowed in patients that failed on ≥ 2 conventional DMARDs. During the full observation period, 91% of patients ever used methotrexate, 85% ever used other conventional DMARDs (systemic glucocorticoids, sulfasalazine, hydroxychloroquine, leflunomide or azathioprine) and 20% ever used biologicals. ACPA-positive patients more frequently used biologicals; further details are shown in Additional file 1: Table S1. According to local and international guidelines, treatment was DAS44 guided with DMARD tapering in case of a DAS < 2.4 and intensifying in case of a DAS ≥ 2.4 [20]. Subsequent to DMARD tapering, DMARDs were stopped in case the DAS44 remained < 2.4 and synovitis was absent at clinical joint examination. Thereafter, patients were followed on the recurrence of synovitis or persistence of DMARD-free remission. The study was approved by the local medical ethics committee, and all patients signed informed consent.

### Sustained DMARD-free remission

Medical files were reviewed for all patients until April 2017 to identify the occurrence of sustained DMARD-free remission, which was defined as the absence of synovitis (by physical examination) that sustained after discontinuation of all DMARD therapy (including biologics and systemic and intra-articular corticosteroids) for the entire follow-up period and must have extended to at least 1 year after DMARD withdrawal. The date of sustained DMARD-free remission was defined as the date 1 year after DMARDs were stopped. Patients who did not achieve remission were censored at the date when the medical file was explored or when they were lost to follow-up. One patient achieved sustained DMARD-free remission but relapsed during follow-up and was considered as not in remission.

### The MBDA score

Serum samples were collected at disease presentation, before any DMARD treatment (including glucocorticoids) was started, and stored at − 80 °C. Crescendo Bioscience (South San Francisco, CA, USA) measured concentrations of 12 biomarkers using three separate multiplex, sandwich immunoassays: CRP, IL-6 (interleukin-6), SAA (serum amyloid A), TNFR1 (tumor necrosis factor receptor superfamily member 1A), EGF (epidermal growth factor), VEGF-A (vascular endothelial growth factor-A), VCAM-1 (vascular cell adhesion molecule-1), MMP-1 (matrix metalloproteinase-1), MMP-3 (matrix metalloproteinase-3), YKL-40 (human cartilage glycoprotein-39), resistin and leptin. Measurements were performed blinded to clinical data and outcome. The biomarkers were studied individually and in combination by using a previously specified algorithm to calculate the MBDA score, ranging on a scale from 1 to 100 [9, 10, 21]. This MBDA algorithm was developed to measure disease activity with DAS28-CRP as reference. For analyses, patients were categorized according to previously established thresholds in categories of low (< 30), moderate (30–44) and high (> 44) MBDA score [10]. Although we used the MBDA score for a purpose different than measuring disease activity, we used the same cut-off points for categorization.

### Statistical analyses

Kaplan-Meier analysis was used to estimate rates of achieving sustained DMARD-free remission with MBDA category and the 12 individual biomarkers as grouping factors. For the latter analyses, patients were categorized into tertiles based on the biomarker levels to create three groups of equal size. Univariable Cox proportional hazards regression analyses were used to assess the association between baseline characteristics and the achievement of sustained DMARD-free remission. Baseline variables with a *p* value < 0.10 were included in a multivariable analysis to assess the independent relation between the serological markers and the achievement of sustained DMARD-free remission. Because achieving sustained DMARD-free remission is mostly confined to ACPA-negative RA and since we aimed to search for subgroups within ACPA-negative and ACPA-positive RA, analyses were stratified for the presence of ACPA. SPSS version 23.0 (IBM) was used. *p* values < 0.05 were considered significant.

## Results

### Patient characteristics

Baseline characteristics of the 299 RA patients are presented in Table 1. The median symptom duration at first presentation was 15 weeks (interquartile range (IQR) 8–32) and, similar to other early arthritis cohorts, 53% of patients were ACPA positive [22, 23].

**Table 1:** Baseline characteristics of all RA patients and of subgroups of ACPA-positive and ACPA-negative patients

	All RA patients (*n* = 299)	ACPA-positive RA patients (*n* = 158)	ACPA-negative RA patients (*n* = 141)
Age in years, mean (SD)	57 (14)	54 (14)	60 (14)
Female, *n* (%)	198 (66)	105 (66)	93 (66)
Symptom duration in weeks, median (IQR)	15 (8–32)	18 (9–38)	12 (5–26)
(Sub)acute symptom onset, *n* (%)	95 (34)	39 (27)	56 (43)
66-SJC, median (IQR)	6 (3–11)	5 (2–8)	8 (3–12)
68-TJC, median (IQR)	9 (4–15)	7 (4–13)	10 (4–18)
RF positivity, *n* (%)	183 (61)	134 (85)	49 (35)
ESR (mm/h), median (IQR)	28 (14–41)	28 (14–41)	28 (11–41)
CRP (μg/mL), median (IQR)	10 (3–23)	8 (3–18)	12 (3–30)
PTGA (0–100), median (IQR)	70 (50–80)	70 (45–80)	70 (60–80)
DAS44, median (IQR)	2.9 (2.4–3.4)	2.8 (2.3–3.3)	3.0 (2.5–3.7)
MBDA category			
Low (< 30), *n* (%)	43 (14)	26 (16)	17 (12)
Moderate (30–44), *n* (%)	64 (21)	35 (22)	29 (21)
High (> 44), *n* (%)	192 (64)	97 (61)	95 (67)

Some data were missing as follows: symptom duration *n* = 4, (sub)acute symptom onset *n* = 23, 66-SJC *n* = 19, 68-TJC *n* = 17, ESR *n* = 3, PTGA *n* = 50 , DAS44 *n* = 20 and CRP *n* = 1

*Symptom duration*, time between symptom onset and inclusion in cohort; *(sub)acute symptom onset*, prompt onset of symptoms (< 1 week); *SD*, standard deviation; *IQR*, interquartile range; *SJC*, 66-swollen joint count; *TJC*, 68-tender joint count; *ESR*, erythrocyte sedimentation rate; *CRP*, C-reactive protein; *PTGA*, patient global assessment; *DAS*, disease activity score; *RF*, rheumatoid factor; *ACPA*, anti-citrullinated protein antibodies; *RA*, rheumatoid arthritis; *MBDA*, multi-biomarker disease activity

### Development of sustained DMARD-free remission and distribution of MBDA scores

The median follow-up duration was 4.3 years (IQR 4.0–4.7). Sustained DMARD-free remission was achieved in 20% (59/299) of RA patients after a median follow-up of 2.9 years (IQR 2.2–4.0). Sustained DMARD-free remission was achieved by 7% (11/158) of ACPA-positive patients and 34% (48/141) of ACPA-negative patients.

### A combination of serological markers as reflected by MBDA scores associated with sustained DMARD-free remission within ACPA-negative RA

**Fig. 1:**
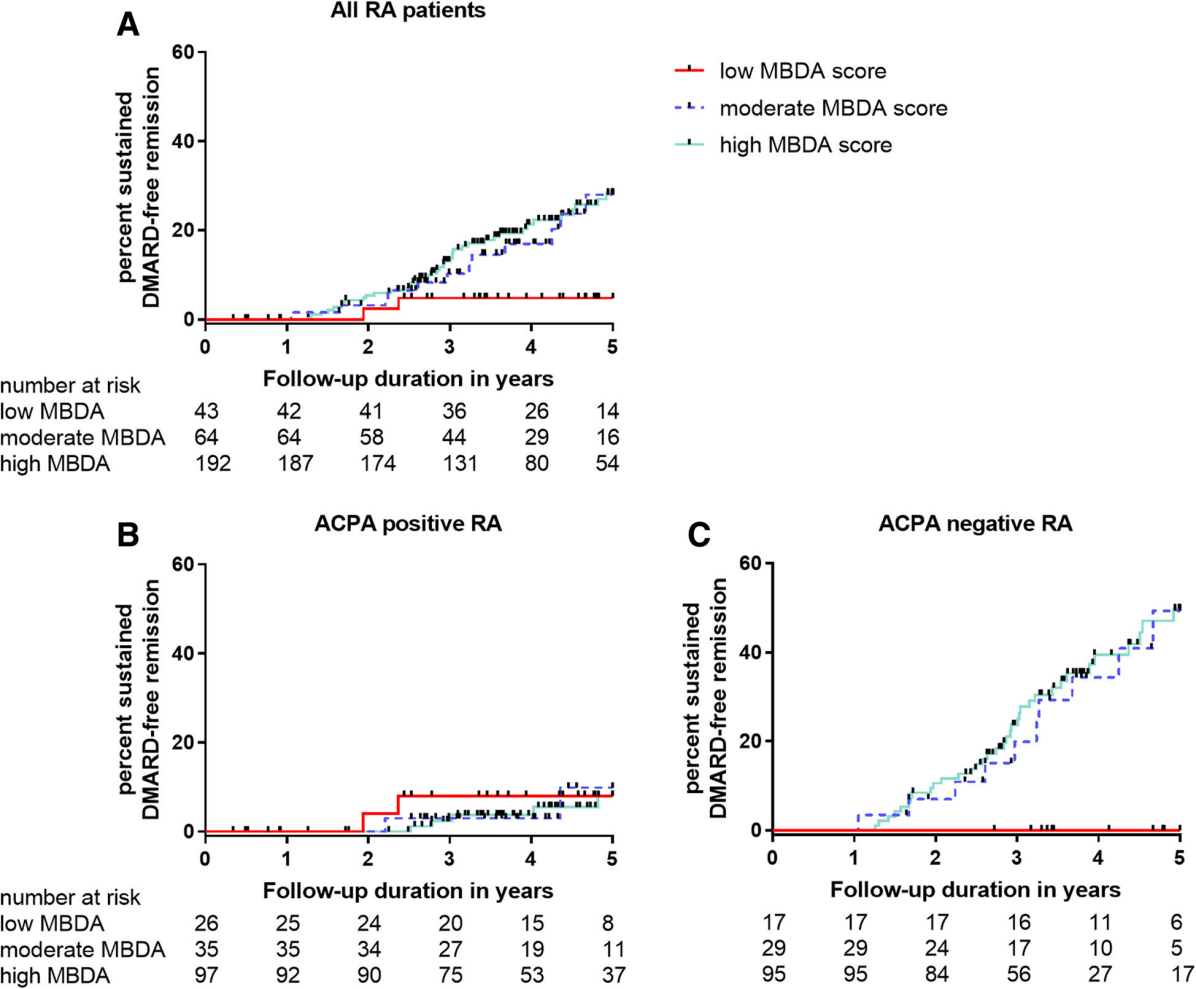
Kaplan-Meier plot showing achievement of sustained DMARD-free remission by category of MBDA score for all RA patients (**a**), ACPA-positive RA patients (**b**) and ACPA-negative (**c**) RA patients. Vertical lines indicate that a patient is censored. The numbers below the figures denote the number of patients at risk in each group. Visual representation of the data was restricted to 5 years follow-up since thereafter the number of patients was small. DMARD, disease-modifying antirheumatic drug; RA, rheumatoid arthritis; ACPA, anti-citrullinated protein antibodies; MBDA, multi-biomarker disease activity

First, the association between the achievement of sustained DMARD-free remission during follow-up and the MBDA score at disease onset was evaluated in all RA patients (Fig. 1a). With patients with low MBDA scores as reference, patients with moderate MBDA scores had an increased probability on the development of sustained DMARD-free remission (hazard ratio (HR) 3.42, 95% confidence interval (CI) 0.97–12.02). A similar increased probability was observed for patients with high MBDA scores (HR 3.79, 95% CI 1.18–12.22). Next, patients were stratified for the presence of ACPA (Fig. 1b, c). For ACPA-positive RA patients, the baseline MBDA category was not associated with achieving sustained DMARD-free remission (moderate vs. low HR 0.75, 95% CI 0.10–8.19; high vs. low HR 0.89, 95% CI 0.19–4.31). By contrast, among ACPA-negative RA patients, moderate or high MBDA scores were strongly associated with achieving sustained DMARD-free remission (moderate vs. low HR 9.40, 95% CI 1.21–72.85; high vs. low HR 9.73 95% CI 1.33–71.10). Sustained DMARD-free remission was almost absent in the ACPA-negative group with low MBDA scores (only one patient in this group achieved remission after 6 years follow-up), whereas sustained DMARD-free remission was achieved by 38% of the ACPA-negative patients with moderate or high MBDA scores. The HR for achieving remission was 9.65 (95% CI 1.33–70.04) when ACPA-negative RA patients with either moderate or high MBDA scores were compared with patients with low MBDA scores. Thus, only for ACPA-negative RA patients, a combination of serological markers at diagnosis, reflected by the MBDA score, was associated with achievement of sustained DMARD-free remission.

### A combination of serological markers associated with sustained DMARD-free remission, independent of clinical factors

Next, we investigated whether the association between baseline MBDA score and sustained DMARD-free remission within ACPA-negative patients was independent of clinical characteristics. Of the clinical baseline characteristics, age at disease onset, the 66-swollen joint count and the presence of RF associated with sustained DMARD-free remission, with a *p* value < 0.10 in ACPA-negative RA; these characteristics were included in a multivariable analysis (Table 2). In this analysis, the MBDA category was associated with sustained DMARD-free remission, independent of these three factors, with moderate vs. low HR 6.96 (95% CI 0.88–55.31) and high vs. low HR 8.19 (95% CI 1.09–61.78) (Table 2).

**Table 2:** Association between the MBDA score and achieving sustained DMARD-free remission over time within ACPA-negative RA patients

	ACPA-negative RA patients					
	Univariable analyses		Multivariable analysis Without MBDA		Multivariable analysis Including MBDA	
	HR (95% CI)	*p* value	HR (95% CI)	*p* value	HR (95% CI)	*p* value
MBDA score						
Low	Reference				Reference	
Moderate	9.40 (1.21–72.85)	0.032			6.96 (0.88–55.31)	0.067
High	9.73 (1.33–71.10)	0.025			8.19 (1.09–61.78)	0.041
Age at disease onset, per year	1.04 (1.02–1.07)	0.001	1.03 (1.01–1.06)	0.006	1.03 (1.00–1.06)	0.036
Female	1.43 (0.80–2.55)	0.23				
Symptom duration > 12 weeks	0.96 (0.54–1.70)	0.89				
(Sub)acute symptom onset	0.93 (0.51–1.69)	0.81				
66-SJC, per joint	1.04 (0.99–1.08)	0.099	1.03 (0.99–1.08)	0.15	1.02 (0.98–1.06)	0.40
68-TJC, per joint	0.99 (0.96–1.02)	0.56				
ESR, per mm/h	1.01 (1.00–1.02)	0.17				
CRP, per µg/mL	1.00 (1.00–1.01)	0.36				
RF positivity	0.57 (0.29–1.09)	0.088	0.84 (0.42–1.66)	0.61	0.78 (0.39–1.58)	0.50

Of the 141 ACPA-negative RA patients, 48 achieved sustained DMARD-free remission. Baseline variables with a *p* value < 0.10 in univariable analyses were included in a multivariable analysis to assess the independent relation between baseline variables and sustained DMARD-free remission

### Among ACPA-negative RA patients, higher CRP, SAA and MMP-3 levels associated with achieving sustained DMARD-free remission

Next, it was studied whether the observed association for ACPA-negative RA patients was driven by a subset of markers of the MBDA score. Therefore, the association between the level of the 12 individual biomarkers included in the MBDA score and the achievement of sustained DMARD-free remission was determined for ACPA-negative RA patients (Additional file 1: Figure S1). Of the individual biomarkers, higher CRP, SAA and MMP-3 levels at disease onset were associated with achieving sustained DMARD-free remission during the follow-up. Patients with CRP levels 7–39 μg/mL (second tertile) had a significantly increased probability on achieving sustained DMARD-free remission compared with patients with CRP levels < 7 μg/mL (lowest tertile) (HR 3.43, 95% CI 1.62–7.27), and for patients with CRP levels ≥ 39 μg/mL (highest tertile), a similar trend was observed (HR 2.12, 95% CI 0.96–4.70). In addition, patients with MMP-3 levels ≥ 60 ng/mL (highest tertile) had a significantly increased probability on the development of sustained DMARD-free remission compared with patients with MMP-3 levels ≤ 28 ng/mL (lowest tertile) (HR 2.18, 95% CI 1.06–4.48). SAA levels were also associated with achieving DMARD-free sustained remission. Patients with SAA levels ≥ 26 μg/mL (highest tertile) or 3–26 μg/mL (second tertile) had a significantly increased probability on the development of sustained DMARD-free remission compared with patients with SAA levels ≤ 3 μg/mL (lowest tertile) (HR 2.87, 95% CI 1.28–6.43 and HR 3.03, 95% CI 1.39–6.63, respectively). The other biomarkers were not individually associated with the achievement of sustained DMARD-free remission.

### Clinical characteristics at disease presentation of ACPA-negative RA patients with an elevated MBDA score

Finally, as ACPA-negative RA patients with a high probability on achieving sustained DMARD-free remission were identifiable by a protein profile that reflected high disease activity at the time of diagnosis, we evaluated whether these patients also had differences in clinical characteristics compared with those presenting with low MBDA scores. ACPA-negative patients with a high MBDA score more often had a subacute onset of symptoms (vs. gradual or intermittent onset) (Table 3). ACPA-negative patients with a moderate or high MBDA score were approximately 10 years older and had higher acute phase reactants at the first presentation, compared with ACPA-negative patients with a low MBDA score (Table 3). These associations with clinical characteristics at diagnosis suggest that subgroups of ACPA-negative RA, differentiated based on serological biomarkers, not only have differences in long-term outcome but also differ already at disease presentation.

**Table 3:** Baseline characteristics of ACPA-negative RA patients per MBDA category

	MBDA category			*p* value
	< 30 (*n* = 17)	30–44 (*n* = 29)	> 44 (*n* = 95)	
Age in years, mean (SD)	48 (16)	60 (13)	62 (14)	< 0.001
Female, *n* (%)	13 (76)	22 (76)	58 (61)	0.21
Symptom duration in weeks, median (IQR)	26 (8–41)	12 (4–25)	12 (5–22)	0.13
(Sub)acute symptom onset, *n* (%)	3 (20)	7 (26)	46 (52)	0.01
66-SJC, median (IQR)	3 (2–7)	6 (3–13)	9 (3–13)	0.08
68-TJC, median (IQR)	12 (9–19)	11 (5–21)	9 (4–17)	0.23
RF positivity, *n* (%)	7 (41)	9 (31)	33 (35)	0.78
ESR (mm/h), median (IQR)	9 (4–14)	14 (6–33)	33 (19–48)	< 0.001
CRP (µg/mL), median (IQR)	3 (3–3)	3 (3–4)	22 (11–44)	< 0.001

Characteristics of ACPA-negative RA patients with low, moderate or high MBDA score were compared with one-way ANOVA, chi-square test and Kruskal-Wallis test, as appropriate

*Symptom duration*, time between symptom onset and inclusion in cohort; *(sub)acute symptom onset*, prompt onset of symptoms (< 1 week); *SD*, standard deviation; *IQR*, interquartile range; *SJC*, 66-swollen joint count; *TJC*, 68-tender joint count; *ESR*, erythrocyte sedimentation rate; *CRP*, C-reactive protein; *RF*, rheumatoid factor; *ACPA*, anti-citrullinated protein antibodies; *RA*, rheumatoid arthritis; *MBDA*, multi-biomarker disease activity

## Discussion

This is the first study showing that ACPA-negative RA patients with a high likelihood of achieving sustained DMARD-free remission during follow-up were identifiable at baseline by a combination of serological markers. This association with sustained DMARD-free remission was independent of clinical baseline characteristics. Furthermore, the ACPA-negative subgroup with a high likelihood of achieving sustained DMARD-free remission showed some differences in clinical characteristics as they were older (mean ≥ 60 years) and more often had a (sub)acute symptom onset. Together, this suggests that a combination of serological biomarkers is helpful in identifying subgroups of ACPA-negative RA patients at disease presentation that differ in baseline characteristics and in their ability to maintain clinical remission after DMARD withdrawal.

Based on differences in genetic and environmental risk factors and in outcome, it is generally accepted that ACPA-positive and ACPA-negative RA are different RA subsets. In the past, we attempted to distinguish subgroups within the group of ACPA-negative RA patients based on only clinical characteristics at disease onset; this did not result in clinically distinguishable subgroups [24]. The current data suggest that a subdivision is possible with serological markers and that, starting from this subdivision, the identified subgroups had some slight differences in clinical characteristics as ACPA-negative RA patients with moderate or high serologic scores at disease onset were older, had more often a (sub)acute onset of symptoms and appeared to have greater inflammatory burden (reflected by higher levels of inflammatory proteins and a tendency towards more swollen joints). Thirty-eight percent of these patients were able to permanently stop DMARDs after a relatively short period of treatment, since DMARD-free remission was achieved after a median disease duration of 2.9 years, which means that DMARDs were stopped after median 1.9 years. Thus, the identified subgroup of ACPA-negative patients was older at disease onset and had more often a prompt onset of symptoms with more severe inflammation but a relative short-term necessity of DMARD treatment. Further studies are needed to confirm these findings.

It is unlikely that ACPA-negative RA patients were misclassified as having RA because patients that during the first year of follow-up were diagnosed with conditions other than RA (e.g. inflammatory osteoarthritis and reactive arthritis) were not included in this study. Also, patients that achieved spontaneous remission, i.e. without the use of DMARDs, were excluded. Patients studied here had a clinical diagnosis of RA and fulfilled classification criteria. In the current taxonomy, these patients are called RA patients. However, our data support the notion that subgroups can be identified within ACPA-negative RA.

A study of established RA patients with a median disease duration of 5 years, who were in sustained remission, showed that high MBDA scores during DMARD treatment and prior to treatment reduction were associated with increased risk of relapses in patients who reduced, and in some cases, stopped, all their DMARD treatments [25]. This might be reflective of subclinical disease activity despite treatment and is conceptually very different from our data. In this study, the MBDA score was used to monitor disease activity, the aim for which the score was derived. In our data, we had a different aim for which measurements were performed in RA patients with very short symptom duration and before any DMARDs were initiated.

High MBDA scores have been associated with radiographic progression in several studies (although most did adjust but not stratify for ACPA) [11–13, 26]. In our study, performed at disease presentation, high MBDA scores strongly associated with a favourable outcome in ACPA-negative RA. This contrasts with the previous findings, but measurements in these studies were done in patients with a disease duration of several years and the studied outcomes were also different.

Our study was focused on achieving sustained DMARD-free remission. Within the group of ACPA-negative RA patients, patients with low MBDA score infrequently achieved this favourable outcome. Numerically, this group was relatively small (12% of ACPA-negative RA patients). Furthermore, this group resembled the ACPA-positive group of RA patients that also infrequently achieved DMARD-free remission. This ACPA-negative subgroup may be interesting for studies on (novel) autoantibody reactivities, as it is speculated that a ‘serological gap’ exists, meaning that part of ACPA-negative patients harbour unmeasured autoantibodies [27]. Moreover, our data revealed that sustained DMARD-free remission is a feasible outcome in about half of the ACPA-negative patients with moderate or high MBDA score.

A limitation is that although rheumatologists at our outpatient clinic are encouraged to try to taper and stop DMARDs in case of DAS remission, patients and rheumatologists were not forced to stop DMARDs if this was felt inappropriate and we did not record how often DMARD tapering was not done despite the presence of DAS remission and the absence of swollen joints. Consequently, the proportion of patients able to achieve sustained DMARD-free remission might be underestimated. It is particularly conceivable that either physicians or patients were reluctant with lowering or stopping medication in the presence of a positive ACPA test.

Another limitation is that the follow-up duration of some patients might have been insufficient to detect flares occurring years after the absence of synovitis, as this may occur after discharge from the outpatient clinic. For this study, patients needed to be in sustained DMARD-free remission for at least 1 year and patients were instructed to return to the outpatient clinic when symptoms recurred, an instruction that is facilitated by the presence of early arthritis recognition clinics and the fact that we are the only referral center in the region [28]. A final limitation is that the number of seronegative patients with low MBDA score was relatively small and therefore (multivariable) analyses within the ACPA-negative subgroup were of limited power resulting in wide confidence intervals of estimated hazard ratios. In addition, resampling methods to show robustness of the data were not performed. Therefore, validation of our results in another early RA cohort is needed.

Remission in this study was defined as the persistent absence of synovitis after DMARD cessation and thus was physician centred. Since synovitis needed to be persistently absent over time, this outcome is different from frequently used remission definitions that are used on single time points. Importantly, we have shown that patients who achieve sustained DMARD-free remission have normalization of functional status and of patient-reported outcomes, underlining that it is the best possible long-term outcome [1].

The MBDA test comprised of serum levels of 12 proteins which were also evaluated separately. Of the different markers, CRP, SAA and MMP-3 were associated with achieving sustained DMARD-free remission. SAA is a protein linked to the acute phase response and is a sensitive indicator of RA disease activity [29, 30]. MMP-3 is a proteinase considered to contribute to cartilage degradation in RA. Its levels have been associated with radiographic progression and also with disease activity and inflammation [31–36]. As the MBDA score was not designed to assess which patients might achieve DMARD-free remission, it is presumable that proteins other than the 12 that were studied here are also differently expressed in subgroups of ACPA-negative RA. Further studies are needed to better characterize this subgroup serologically. Additionally, biologic studies are needed to identify pathways that are relevant for the development of this subgroup of RA patients.

## Conclusions

In conclusion, ACPA-negative RA patients who achieved sustained DMARD-free remission during follow-up were characterized by differences in protein expression at disease presentation. This is the first evidence that ACPA-negative RA can be subdivided at disease onset in clinically relevant subgroups with differences in the likelihood of achieving and maintaining clinical remission after treatment withdrawal.

## Additional file

Additional file 1: **Table S1.** Overview of medication used by all RA patients and by the subgroups of ACPA-positive and ACPA-negative RA patients during the total follow-up duration. **Figure S1.** Kaplan-Meier plots showing achievement of sustained DMARD-free remission by ACPA-negative RA patients (*n* = 141) grouped by tertiles of 12 serum biomarkers measured at disease presentation. (DOCX 305 kb)

## Abbreviations

ACPA: Anti-citrullinated protein antibodies
CI: Confidence interval
CRP: C-reactive protein
DAS: Disease activity score
DMARD: Disease-modifying antirheumatic drug
EGF: Epidermal growth factor
ESR: Erythrocyte sedimentation rate
HR: Hazard ratio
IL-6: Interleukin-6
IQR: Interquartile range
MBDA: Multi-biomarker disease activity
MMP-1: Matrix metalloproteinase-1
MMP-3: Matrix metalloproteinase-3
RA: Rheumatoid arthritis
RF: Rheumatoid factor
SAA: Serum amyloid A
TNFR1: Tumor necrosis factor receptor superfamily member 1A
VCAM-1: Vascular cell adhesion molecule-1
VEGF-A: Vascular endothelial growth factor-A
YKL-40: Human cartilage glycoprotein-39

## References

[1] de Rooy DPC, van der Linden MPM, Knevel R, TWJ H, van der H Mil AHM (2011). Predicting arthritis outcomes—what can be learned from the Leiden Early Arthritis Clinic?. Rheumatology..

[2] van der Woude D, Young A, Jayakumar K, Mertens BJ, Toes REM, van der Heijde D (2009). Prevalence of and predictive factors for sustained disease-modifying antirheumatic drug-free remission in rheumatoid arthritis: results from two large early arthritis cohorts. Arthritis Rheum.

[3] van der Linden MPM, le Cessie S, Raza K, van der Woude D, Knevel R, Huizinga TWJ (2010). Long-term impact of delay in assessment of patients with early arthritis. Arthritis Rheum.

[4] van Nies JAB, Tsonaka R, Gaujoux-Viala C, Fautrel B, van der Helm-van Mil AHM (2015). Evaluating relationships between symptom duration and persistence of rheumatoid arthritis: does a window of opportunity exist? Results on the Leiden early arthritis clinic and ESPOIR cohorts. Ann Rheum Dis.

[5] van Nies JAB, Krabben A, Schoones JW, Huizinga TWJ, Kloppenburg M, van der Helm-van Mil AHM (2014). What is the evidence for the presence of a therapeutic window of opportunity in rheumatoid arthritis? A systematic literature review. Ann Rheum Dis.

[6] van der Kooij SM, Goekoop-Ruiterman YPM, de Vries-Bouwstra JK, Güler-Yüksel M, Zwinderman AH, Kerstens PJSM (2009). Drug-free remission, functioning and radiographic damage after 4 years of response-driven treatment in patients with recent-onset rheumatoid arthritis. Ann Rheum Dis.

[7] van der Woude D, Visser K, Klarenbeek NB, Ronday HK, Peeters AJ, Kerstens PJSM (2012). Sustained drug-free remission in rheumatoid arthritis after DAS-driven or non-DAS-driven therapy: a comparison of two cohort studies. Rheumatol Oxf Engl..

[8] Ajeganova S, van Steenbergen HW, van Nies JAB, Burgers LE, Huizinga TWJ, van der Helm-van Mil AHM (2016). Disease-modifying antirheumatic drug-free sustained remission in rheumatoid arthritis: an increasingly achievable outcome with subsidence of disease symptoms. Ann Rheum Dis.

[9] Curtis JR, van der Helm-van Mil AH, Knevel R, Huizinga TW, Haney DJ, Shen Y (2012). Validation of a novel multibiomarker test to assess rheumatoid arthritis disease activity. Arthritis Care Res.

[10] Centola M, Cavet G, Shen Y, Ramanujan S, Knowlton N, Swan KA (2013). Development of a multi-biomarker disease activity test for rheumatoid arthritis. PLoS One.

[11] Hambardzumyan K, Bolce R, Saevarsdottir S, Cruickshank SE, Sasso EH, Chernoff D (2015). Pretreatment multi-biomarker disease activity score and radiographic progression in early RA: results from the SWEFOT trial. Ann Rheum Dis.

[12] Hambardzumyan K, Bolce RJ, Saevarsdottir S, Forslind K, Wallman JK, Cruickshank SE (2016). Association of a multibiomarker disease activity score at multiple time-points with radiographic progression in rheumatoid arthritis: results from the SWEFOT trial. RMD Open.

[13] Markusse IM, Dirven L, van den Broek M, Bijkerk C, Han KH, Ronday HK (2014). A multibiomarker disease activity score for rheumatoid arthritis predicts radiographic joint damage in the BeSt study. J Rheumatol.

[14] Bouman CAM, van der Maas A, van Herwaarden N, Sasso EH, van den Hoogen FHJ, den Broeder AA (2017). A multi-biomarker score measuring disease activity in rheumatoid arthritis patients tapering adalimumab or etanercept: predictive value for clinical and radiographic outcomes. Rheumatol Oxf Engl..

[15] Bakker MF, Cavet G, Jacobs JW, Bijlsma JWJ, Haney DJ, Shen Y (2012). Performance of a multi-biomarker score measuring rheumatoid arthritis disease activity in the CAMERA tight control study. Ann Rheum Dis.

[16] Fleischmann R, Connolly SE, Maldonado MA, Schiff M (2016). Brief report: estimating disease activity using multi-biomarker disease activity scores in rheumatoid arthritis patients treated with abatacept or adalimumab. Arthritis Rheumatol.

[17] van der Linden MPM, Batstra MR, Bakker-Jonges LE, Foundation for Quality Medical Laboratory Diagnostics, Detert J, Bastian H, et al. Toward a data-driven evaluation of the 2010 American College of Rheumatology/European League Against Rheumatism criteria for rheumatoid arthritis: is it sensible to look at levels of rheumatoid factor? Arthritis Rheum 2011;63:1190–1199. 10.1002/art.30200 21538311

[18] Arnett FC, Edworthy SM, Bloch DA, Mcshane DJ, Fries JF, Cooper NS (1988). The American Rheumatism Association 1987 revised criteria for the classification of rheumatoid arthritis. Arthritis Rheum.

[19] Aletaha D, Neogi T, Silman AJ, Funovits J, Felson DT, Bingham CO (2010). 2010 rheumatoid arthritis classification criteria: an American College of Rheumatology/European League Against Rheumatism collaborative initiative. Ann Rheum Dis.

[20] Smolen JS, Landewé R, Bijlsma J, Burmester G, Chatzidionysiou K, Dougados M (2017). EULAR recommendations for the management of rheumatoid arthritis with synthetic and biological disease-modifying antirheumatic drugs: 2016 update. Ann Rheum Dis.

[21] Eastman PS, Manning WC, Qureshi F, Haney D, Cavet G, Alexander C (2012). Characterization of a multiplex, 12-biomarker test for rheumatoid arthritis. J Pharm Biomed Anal.

[22] Ajeganova S, Humphreys JH, Verheul MK, van Steenbergen HW, van Nies JAB, Hafström I (2016). Anticitrullinated protein antibodies and rheumatoid factor are associated with increased mortality but with different causes of death in patients with rheumatoid arthritis: a longitudinal study in three European cohorts. Ann Rheum Dis.

[23] Roubille C, Rincheval N, Dougados M, Flipo R-M, Daurès J-P, Combe B (2017). Seven-year tolerability profile of glucocorticoids use in early rheumatoid arthritis: data from the ESPOIR cohort. Ann Rheum Dis.

[24] De Rooy DPC, Willemze A, Mertens B, Huizinga TWJ, Van der Helm-van Mil AHM (2011). Can anti-cyclic citrullinated peptide antibody-negative RA be subdivided into clinical subphenotypes?. Arthritis Res Ther..

[25] Rech J, Hueber AJ, Finzel S, Englbrecht M, Haschka J, Manger B (2016). Prediction of disease relapses by multibiomarker disease activity and autoantibody status in patients with rheumatoid arthritis on tapering DMARD treatment. Ann Rheum Dis.

[26] Li W, Sasso EH, van der Helm-van Mil AHM, Huizinga TWJ (2016). Relationship of multi-biomarker disease activity score and other risk factors with radiographic progression in an observational study of patients with rheumatoid arthritis. Rheumatology (Oxford).

[27] Trouw LA, Mahler M (2012). Closing the serological gap: promising novel biomarkers for the early diagnosis of rheumatoid arthritis. Autoimmun Rev.

[28] van Nies JAB, Brouwer E, van Gaalen FA, Allaart CF, Huizinga TWJ, Posthumus MD (2013). Improved early identification of arthritis: evaluating the efficacy of Early Arthritis Recognition Clinics. Ann Rheum Dis.

[29] Cunnane G, Grehan S, Geoghegan S, McCormack C, Shields D, Whitehead AS (2000). Serum amyloid A in the assessment of early inflammatory arthritis. J Rheumatol.

[30] Shen C, Sun X-G, Liu N, Mu Y, Hong C-C, Wei W (2015). Increased serum amyloid A and its association with autoantibodies, acute phase reactants and disease activity in patients with rheumatoid arthritis. Mol Med Rep.

[31] Shiozawa K, Yamane T, Murata M, Yoshihara R, Tsumiyama K, Imura S (2016). MMP-3 as a predictor for structural remission in RA patients treated with MTX monotherapy. Arthritis Res Ther..

[32] Ma J-D, Wei X-N, Zheng D-H, Mo Y-Q, Chen L-F, Zhang X (2015). Continuously elevated serum matrix metalloproteinase-3 for 3 ~ 6 months predict one-year radiographic progression in rheumatoid arthritis: a prospective cohort study. Arthritis Res Ther.

[33] Yamanaka H, Matsuda Y, Tanaka M, Sendo W, Nakajima H, Taniguchi A (2000). Serum matrix metalloproteinase 3 as a predictor of the degree of joint destruction during the six months after measurement, in patients with early rheumatoid arthritis. Arthritis Rheum.

[34] Hiura K, Iwaki-Egawa S, Kawashima T, Fujisawa S-I, Takeda T, Komori H (2013). The diagnostic utility of matrix metalloproteinase-3 and high-sensitivity C-reactive protein for predicting rheumatoid arthritis in anti-cyclic citrullinated peptide antibody-negative patients with recent-onset undifferentiated arthritis. Rheumatol Int.

[35] Green MJ, Gough AKS, Devlin J, Smith J, Astin P, Taylor D (2003). Serum MMP-3 and MMP-1 and progression of joint damage in early rheumatoid arthritis. Rheumatol Oxf Engl..

[36] Posthumus MD, Limburg PC, Westra J, van Leeuwen MA, van Rijswijk MH (2000). Serum matrix metalloproteinase 3 in early rheumatoid arthritis is correlated with disease activity and radiological progression. J Rheumatol.

