# Supplementary material for: ACPA-negative RA consists of subgroups: patients with high likelihood of achieving sustained DMARD-free remission can be identified by serological markers at disease presentation
Source: Arthritis Res Ther. 2019 May 14;21:121. doi: 10.1186/s13075-019-1902-2 (PMC6518725; doi:10.1186/s13075-019-1902-2)
Supplement: Supplementary file 1 — Table S1. Overview of medication used by all RA patients and by the subgroups of ACPA-positive and ACPA-negative RA patients during the total follow-up duration. Figure S1. Kaplan-Meier plots showing achievement of sustained DMARD-free remission by ACPA-negative RA patients (n = 141) grouped by tertiles of 12 serum biomarkers measured at disease presentation. (DOCX 305 kb) [file 13075_2019_1902_MOESM1_ESM.docx]

**Table S1 Overview of medication used by all RA-patients and by the subgroups of ACPA-positive and ACPA-negative RA-patients during the total follow-up duration**

|  | | All RA-patients (n=299) | Subgroup of ACPA-positive RA-patients (n=158) | Subgroup of ACPA-negative RA-patients (n=141) |
| --- | --- | --- | --- | --- |
| Methotrexate, n(%) | | 271 (91) | 152 (96) | 119 (84) |
| Other conventional DMARDs, n(%) | | 254 (85) | 140 (89) | 114 (81) |
|  | Systemic glucocorticoids, n(%) | 228 (76) | 127 (80) | 101 (72) |
|  | Sulfasalazine, n(%) | 102 (34) | 61 (39) | 41 (29) |
|  | Hydroxychloroquine, n(%) | 131 (44) | 88 (56) | 43 (30) |
|  | Leflunomide, n(%) | 56 (19) | 46 (29) | 10 (7) |
|  | Azathioprine, n(%) | 6 (2) | 3 (2) | 3 (2) |
| Biological DMARD, n(%) | | 60 (20) | 48 (30) | 12 (9) |
|  | TNF-inhibitor, n(%) | 48 (16) | 39 (25) | 9 (6) |
|  | Rituximab, n(%) | 3 (1) | 2 (1) | 1 (0.7) |
|  | Abatacept, n(%) | 5 (2) | 5 (3) | 0 (0) |
|  | Tocilizumab, n(%) | 16 (5) | 14 (9) | 2 (1) |
|  | Omalizumab, n(%) | 5 (2) | 5 (3) | 0 (0) |
|  | Baricitinib, n(%) | 1 (0.3) | 1 (0.6) | 0 (0) |

Numbers indicate the number of patients that used the indicated medication at any time during follow-up. The number of patients using combination therapy, or of patients using several medications during follow-up is not specified here. RA, rheumatoid arthritis; ACPA, anti-citrullinated protein antibodies; DMARD, disease-modifying antirheumatic drug; TNF, tumor necrosis factor.

**
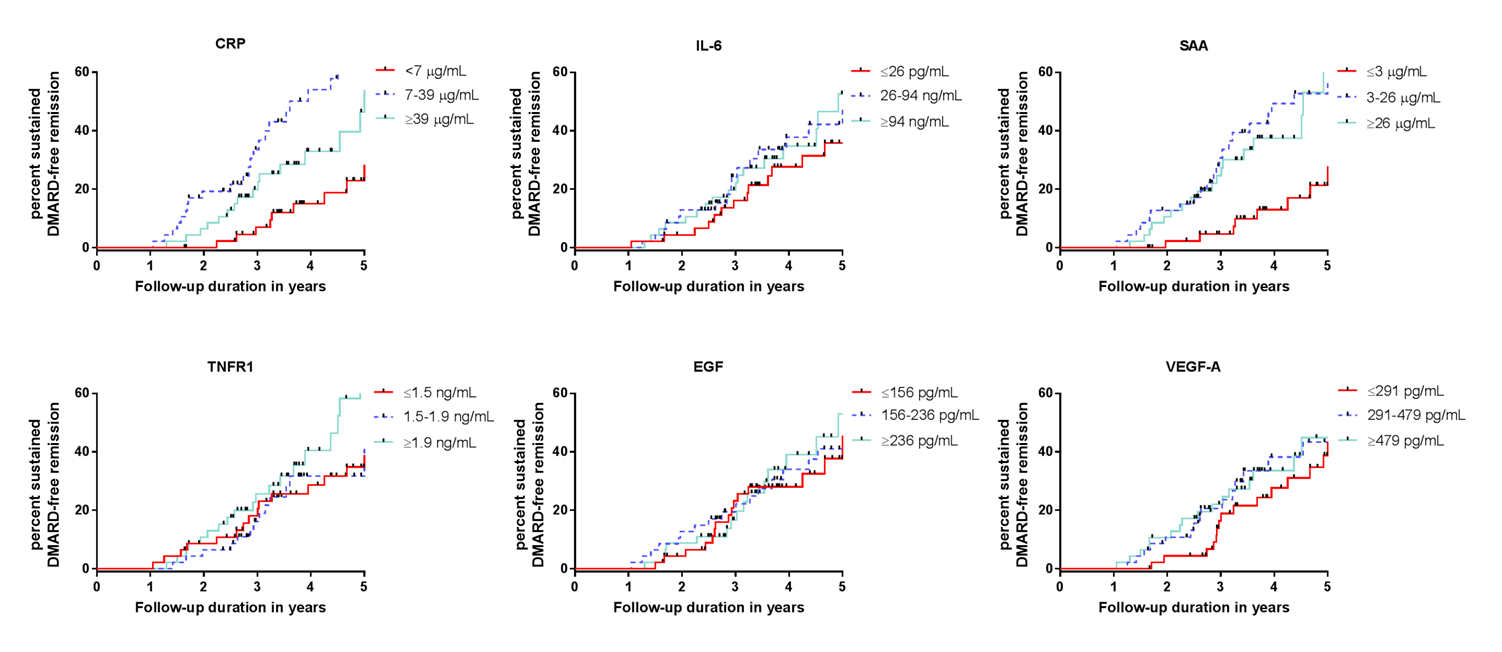
Figure S1 Kaplan Meier plots showing achievement of sustained DMARD-free remission by ACPA-negative RA-patients (n=141) grouped by tertiles of 12 serum biomarkers measured at disease presentation**

**
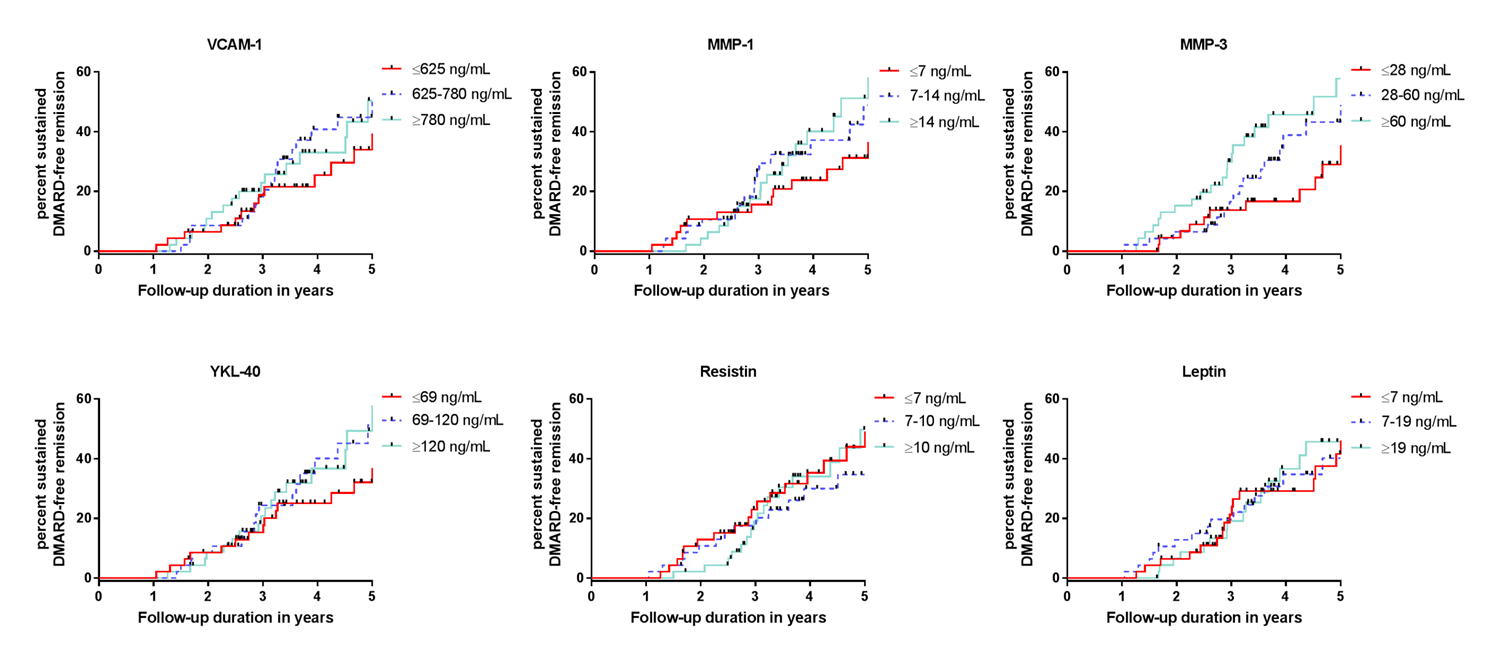
**

Higher CRP, SAA and MMP-3 levels were associated with achieving sustained DMARD-free remission. The other biomarkers were not associated with remission. Patients were categorized into tertiles based on the biomarker levels to create three groups of equal size. Vertical lines indicate that a patient is censored. DMARD, disease-modifying antirheumatic drug; MBDA, multi-biomarker disease activity; CRP, C-reactive protein; IL-6, interleukin 6; SAA, serum amyloid A; TNFR1, tumor necrosis factor receptor superfamily member 1A; EGF, epidermal growth factor; VEGF-A, vascular endothelial growth factor A; VCAM-1, vascular cell adhesion molecule 1; MMP-1, matrix metalloproteinase 1; MMP-3, matrix metalloproteinase 3; YKL-40, human cartilage glycoprotein 39.
